# Assessment of body composition by whole-body densitometry: what radiologists should know

**DOI:** 10.1590/0100-3984.2021.0155-en

**Published:** 2022

**Authors:** Lucas Gabriel Cruz de Menezes Chaves, Thiago José Martins Gonçalves, Almir Galvão Vieira Bitencourt, Ricardo Arroyo Rstom, Talita Rombaldi Pereira, Silvio Fontana Velludo

**Affiliations:** 1 Instituto Prevent Senior, São Paulo, SP, Brazil; 2 A.C.Camargo Cancer Center, São Paulo, SP, Brazil

**Keywords:** Densitometry, Body composition, Body fat distribution, Sarcopenia, Densitometria, Composição corporal, Distribuição da gordura corporal, Sarcopenia

## Abstract

The analysis of body composition is a fundamental part of a nutritional status assessment, and the use of diagnostic imaging methods has been increasingly required for an adequate characterization of the lean body mass and fat mass. Body composition measurements are useful in evaluating the effectiveness of nutritional interventions and monitoring changes associated with aging and chronic diseases. Whole-body densitometry using dual-energy X-ray absorptiometry (DEXA) is one of the most widely used methods in clinical practice, allowing highly accurate assessment of the bone mineral content, lean body mass, and fat mass. Although a DEXA examination provides a lot of information, there is still no universal standardization of the parameters to be included in radiology reports. The aim of this study was to review the most relevant information for assessing body composition by whole-body densitometry.

## Abstract (pt)

A análise da composição corporal é parte fundamental de uma avaliação nutricional, em que a utilização de métodos diagnósticos por imagem tem sido cada vez mais requisitada para uma adequada caracterização da massa magra e massa gorda corporal. Medidas de composição corporal são úteis em avaliar a eficácia das intervenções nutricionais e monitorar as mudanças associadas ao envelhecimento e condições de doenças crônicas. A densitometria de corpo inteiro utilizando a técnica de absorciometria de dupla energia (DEXA) é um dos métodos mais utilizados na prática clínica, que permite avaliação com elevada acurácia do conteúdo mineral ósseo, da gordura corporal e da massa magra. Este exame fornece grande quantidade de informações, no entanto, ainda não existe uma padronização universal de quais parâmetros devem ser incluídos nos relatórios radiológicos. O objetivo deste trabalho é revisar as informações mais relevantes para avaliação da composição corporal fornecidas pela densitometria de corpo inteiro.

## INTRODUCTION

The human body is primarily composed of four molecular-level components-water, fat, protein, and minerals-typically in descending order by quantity. Body composition analysis is a fundamental part of a nutritional assessment, allowing the accurate diagnosis of conditions such as visceral obesity, as well as being useful in the diagnostic investigation of sarcopenia, which may be related to higher risk and worse prognosis in various types of clinically and surgically treated diseases. In addition, changes in body composition are known to be associated with several diseases, such as cardiovascular diseases, diabetes, cancer, osteoporosis, and osteoarthritis^(1)^.

Anthropometric parameters-including body mass index, waist circumference, and waist-to-hip ratio, and skinfold thickness-have been used for the indirect assessment of body composition in clinical practice, although those measures have limitations, especially in patients who are elderly or obese^(2,3)^. Another technique routinely used in clinical practice is bioelectrical impedance, which is quite accessible and affordable, allowing the evaluation of multiple parameters such as total body water, fat mass, and lean body mass. However, that technique also has limitations, related to the variation in results among different devices, as well as inter- and intra-individual variability, which can be attributed to nutritional status, hydration, physical activity, diet, age, and comorbidities^(3-6)^.

When comparing methods of body composition analysis, it is important to distinguish fat mass from adipose tissue, which is approximately only fat mass, the remainder being water, protein, and minerals. Although most body fat is stored in adipose tissue, fat is also present in organs such as the liver (potentially leading to hepatic steatosis) and skeletal muscle (potentially leading to myosteatosis). It is now well known that the metabolic risk related to fat accumulation is strongly dependent on its distribution within the body^(1,2,7)^. In addition to fat, which functions as long-term energy storage, the study of skeletal muscle mass is of great interest in clinical practice, and knowledge of the balance between the energy consumed by muscles and that stored in fat compartments is therefore highly relevant to the understanding of metabolic balance^(1)^.

Imaging methods have been increasingly used in order to facilitate the evaluation of body composition, as well as the monitoring of the different body compartments and their distribution, allowing the appropriate characterization of lean body mass and fat mass. Several imaging methods have been studied, including ultrasound, magnetic resonance imaging (MRI), computed tomography (CT), and whole-body densitometry using the dual-energy X-ray absorptiometry (DEXA) technique, the last two being the most commonly used in clinical practice. Due to the high radiation dose, the use of CT for body composition assessment is reserved for patients who are undergoing CT for another clinical indication (“convenience imaging”). The main advantages of DEXA include the fact that it is a rapid method, is widely available, and is affordable; it allows a highly accurate assessment of bone mineral content, fat mass, and lean body mass, with well-established reference values^(7-11)^.

The analysis of body composition, especially the estimation of bone mineral content and total body fat, has been shown to be more accurate with DEXA than with other body density-based methods^(12-15)^. Although DEXA assumes constant hydration of the lean body mass, it should be borne in mind that hydration varies depending on the age and sex of the patient, as well as in the presence of a chronic disease, which could be a limitation in some specific groups of patients, especially elderly patients with comorbidities^(12,13)^. Therefore, the degree of hydration can be a confounding factor in the assessment of lean mass by DEXA and variations in hydration must be taken into account in the analysis of changes related to nutritional interventions or physical activity over time, especially in athletes^(14,15)^.

Although DEXA has been used with increasing frequency in clinical practice, there is still no standardization specific for reports of densitometry tests of body composition in Brazil. The objective of this article is to review the most relevant information provided by DEXA for the assessment of body composition.

## INDICATIONS FOR DEXA

According to the International Society of Clinical Densitometry^(12)^, the main indications for the assessment of body composition by DEXA are as follows: to assess lean body mass and fat mass in patients who have been treated for obesity, either clinically (with diet or medications) or surgically (with bariatric surgery), and who have achieved a ≥ 10% weight loss; to quantify appendicular lean body mass in patients at risk for sarcopenia and in patients presenting with muscle weakness or poor physical performance; and to assess body fat, because of the risk of lipodystrophy, in HIV-infected patients on antiretroviral therapy (with zidovudine or stavudine). A DEXA body composition assessment may also be indicated for athletes or any individual as part of the assessment of nutritional status, as well as to monitor the results of weight loss interventions such as diet, physical activity, and drug treatment^(12)^.

According to the latest update of the consensus statement issued by the European Working Group on Sarcopenia in Older People^(16)^, sarcopenia is defined as a syndrome characterized by progressive, generalized loss of muscle strength, together with a quantitative or qualitative loss of skeletal muscle mass, which is associated with adverse events and worse clinical outcomes, such as a loss of physical independence and reduced quality of life, as well as an increased risk of falls/fractures and death. Sarcopenia can be primary, when associated with the aging process, or secondary, when associated with other triggers, such as inadequate protein intake, gastrointestinal malabsorption disorders, critical illness, cancer, and various chronic diseases (e.g., chronic kidney disease, chronic obstructive pulmonary disease, and severe congestive heart failure). The current consensus recommends that the diagnosis of sarcopenia be based on a number of factors^(7,16)^: reduced muscle strength alone is indicative of probable sarcopenia; the diagnosis of sarcopenia can be confirmed if there is also a low quantity/quality of skeletal muscle mass; and patients who progress to poor physical performance are categorized as having severe sarcopenia.

## PREPARATION FOR AND TECHNIQUE EMPLOYED IN DEXA

It is necessary to differentiate between a physician order for DEXA in which the objective is the assessment of body composition and one in which the objective is conventional bone densitometry. Although both tests are performed in the same scanner, bone densitometry, which is routinely used for the diagnosis and monitoring of osteopenia and osteoporosis, evaluates specific bone sites, including the lumbar spine, proximal femur, and forearm. The evaluation of body composition by DEXA requires a whole-body scan, which is not routinely performed to assess bone density in adults (except in those under 20 years of age).

Although there is no specific preparation for DEXA, the examination should not be performed after any other imaging examination in which contrast was administered (e.g., contrast-enhanced X-ray or CT scan), especially one in which oral contrast was used. Because it uses ionizing radiation, DEXA is contraindicated in pregnant women. It also cannot be performed if the weight of the patient exceeds the capacity of the equipment, which ranges from 160 kg to 225 kg among devices. The dose of ionizing radiation employed in DEXA is quite low (0.001 mSv), approximately one tenth of that employed in a simple chest X-ray. In DEXA, the X-ray source generates a dual-energy beam that is attenuated during its passage through the body, being influenced by the intensity of the energy, as well as by the density and thickness of the tissues^(17,18)^.

**Figure 1:**
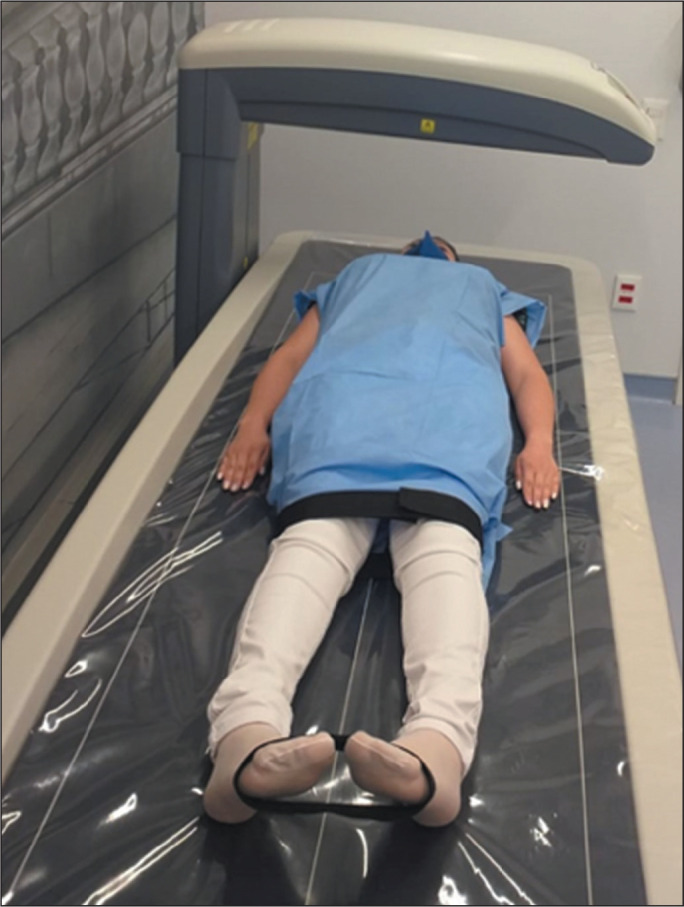
Patient positioned for whole-body densitometry to assess body composition.

**Figure 2:**
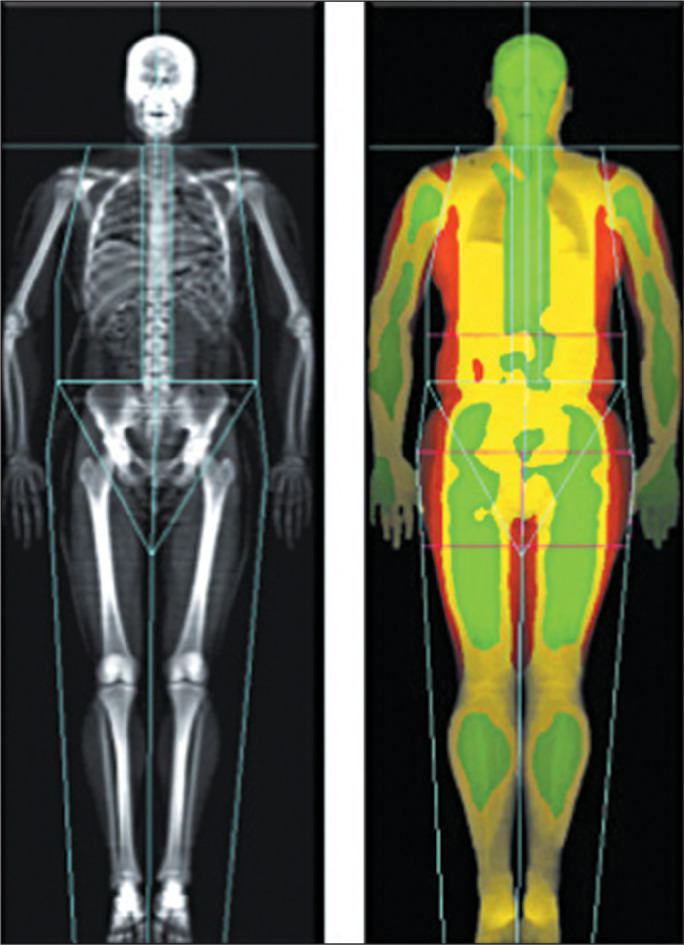
Example of DEXA performed to assess body composition, with proper positioning of the reference lines separating the regions of the body: head, trunk, pelvis, arms, and legs.

A DEXA examination is performed with the patient in the supine position (Figure 1), with conventional densitometry equipment (the same used for bone densitometry examinations), and takes 2-10 min, the examination time varying depending on the scanner used and the size of the patient. However, it is still possible to assess body composition by region (trunk, arms, and legs) through proper positioning of the reference lines (Figure 2). For patients who are very tall or very wide, in whom it is not possible to acquire a whole-body scan in a single acquisition, the devices rely on specific mirroring techniques that allow the “reconstruction” of the image of a limb based on the image of the contralateral limb, for example^(8,11)^.

## ANALYSIS AND INTERPRETATION OF DEXA IMAGES

The various components of body composition can be analyzed by DEXA, which can identify differences in density among bone mineral content, lean body mass, and fat mass. Bone mineral density values obtained from whole-body densitometry are not used for the diagnosis of osteopenia or osteoporosis in adults, which requires a targeted bone densitometry examination to assess specific sites (the lumbar spine, proximal femur, and forearm). Therefore, in a DEXA examination performed for the evaluation of body composition, the bone mineral content data are less important than are the lean body mass and fat mass data. Table 1 summarizes the lean and fat mass data obtained with DEXA.

**Table 1:** DEXA examination variables for determining body composition, with reference values.

Measure	Variable	Reference value
kg	Fat mass	-
%	Body fat percentage	-
kg/m^2^	FMI	3-6 for men; 5-9 for women
-	Android/genoid fat ratio	< 1
cm^2^	Visceral adipose tissue (VAT)	< 100
cm^2^	Subcutaneous adipose tissue (SAT)	-
-	VAT/SAT ratio	< 0.4
kg/m^2^	Appendicular lean mass index (ALMI)	> 7 for men; > 5.5 for women

**Figure 3:**
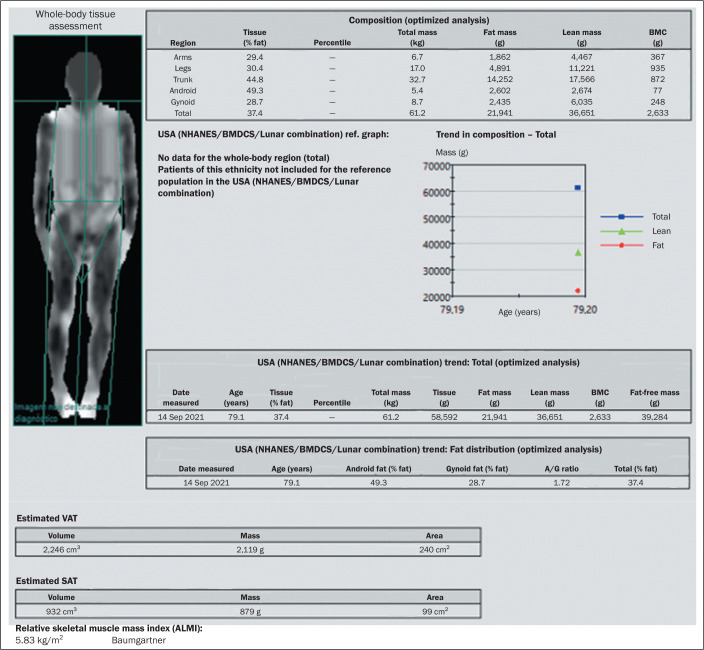
Example of DEXA in a 79-year-old male patient with a low body mass index (22.7 kg/m^2^; weight: 61 kg ; height: 1.64 cm). Body fat analysis revealed a fat mass of 21.9 kg (body fat: 35.8%), with a FMI of 8.1 kg/m^2^ (consistent with overweight), a VAT area of 240 cm^2^ (normal range, ≤ 100 cm^2^), an android/gynoid (A/G) fat ratio of 1.72 (predominance of android fat), and an ALMI of 5.8 kg/m^2^ (ALM: 15.7 kg, which is considered low).

**Figure 4:**
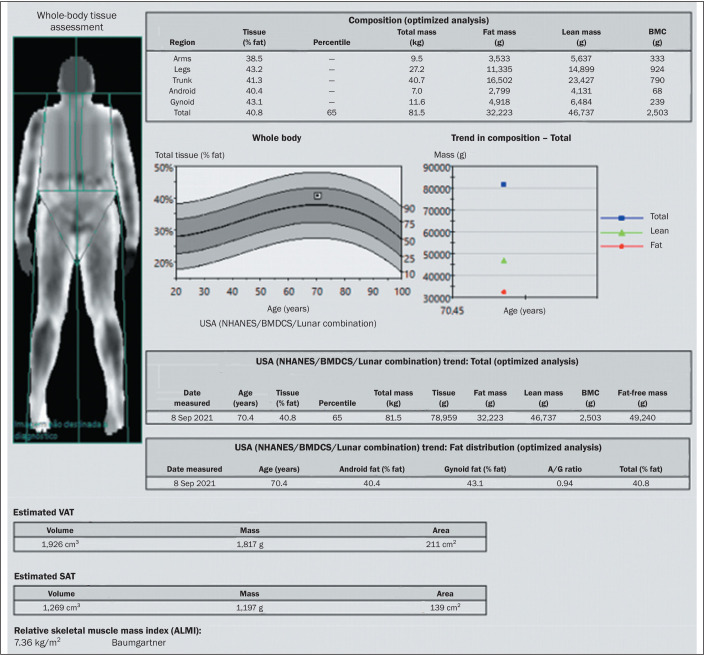
Example of DEXA in a 70-year-old female patient with a low body mass index (21.9 kg/m^2^; weight: 61 kg; height: 1.67 cm). Body fat analysis revealed a fat mass of 32.2 kg (body fat: 39.6%), with a FMI of 11.5 kg/m^2^ (consistent with overweight), a VAT area of 211 cm^2^ (normal range, ≤ 100 cm^2^), an android/gynoid (A/G) fat ratio of 0.94 (predominance of gynoid fat), and an ALMI of 7.4 kg/m^2^ (ALM: 20.5 kg, which is considered normal).

The use of DEXA allows adipose tissue to be detected with high accuracy, making it possible to calculate the percentage of body fat and the fat mass index (FMI), as well as the android/gynoid fat ratio (Figures 3 and 4). Unlike the body mass index, which is based on total body weight, the FMI is based on body fat only and has well-established reference values for both sexes^(19)^, therefore being considered a better tool for the assessment of overweight and obesity (Table 2).

**Table 2:** Reference values for the FMI.

Categories	FMI reference values	
	Men	Women
Class III obesity	> 15.0 kg/m^2^	> 21.0 kg/m^2^
Class II obesity	12.1-15.0 kg/m^2^	17.1-21.0 kg/m^2^
Class I obesity	9.1-12.0 kg/m^2^	13.1-17.0 kg/m^2^
Overweight	6.1-9.0 kg/m^2^	9.1-13.0 kg/m^2^
Normal weight	3-6 kg/m^2^	5-9 kg/m^2^
Mild fat deficit	2.3-3.0 kg/m^2^	4.0-4.9 kg/m^2^
Moderate fat deficit	2.0-2.2 kg/m^2^	3.5-3.9 kg/m^2^
Marked fat deficit	< 2.0 kg/m^2^	< 3.5 kg/m^2^

Source: Adapted from Kelly et al.^(^8^)^.

BMC, bone mineral content.

BMC, bone mineral content.

Gynoid fat, also known as peripheral or gluteofemoral fat, is concentrated in the pelvis and thighs and is associated with a lower cardiovascular risk than is android fat, also known as central or truncal fat, which is concentrated in the abdominal region and is associated with a higher risk of metabolic complications. Therefore, an android/gynoid fat ratio greater than 1 (android fat predominance) increases the risk of cardiovascular disease, dyslipidemia, insulin resistance, type 2 diabetes, and metabolic syndrome^(20)^.

Modern DEXA devices also allow the quantification of visceral adipose tissue (VAT) and subcutaneous adipose tissue (SAT) in the abdominal region. The measurement of VAT is traditionally performed with axial imaging methods, such as CT and MRI, either by volumetric evaluation or by assessing the area of VAT in an axial slice, most commonly acquired at the L3 level^(21)^. The DEXA-based estimation of the VAT area has been found to correlate well with the CT-based estimation and has been routinely used in the assessment of body composition^(22-24)^. A VAT area ≥ 100 cm^2^ is associated with high cardiovascular risk, whereas a VAT area ≥ 160 cm^2^ is associated with very high cardiovascular risk^(25)^. In addition, the VAT/SAT ratio provides a relative index for the accumulation of abdominal fat, a VAT/SAT ratio ≥ 0.4 (predominance of VAT) being a major risk factor for disorders of glucose and lipid metabolism^(26)^.

The assessment of lean body mass can be used for the diagnosis and monitoring of sarcopenia. With DEXA, body composition can be assessed with good accuracy and low radiation exposure. However, it should be borne in mind that although DEXA assesses overall lean body mass, it does not assess skeletal muscle mass separately. The lean body mass evaluated by DEXA includes skeletal muscle mass, viscera, and fluids, which have similar radiological density. Therefore, it is not possible to differentiate among them in the densitometry examination. Consequently, the measure used for the analysis of lean body mass in DEXA is the ALM; that is, the sum of the lean body mass of the arms and legs, excluding the trunk region where there is greater overlap with viscera and liquids. Because DEXA does not assess skeletal muscle mass directly, some physicians are reluctant to accept it as the gold standard for this purpose. Despite those limitations, specific cutoff points have been proposed for specific populations, with the aim of identifying low muscle mass, using the ALMI devised by Baumgartner et al.^(27)^. The ALMI is calculated as the ALM divided by the height in meters squared. In the investigation of sarcopenia, a diagnosis of low lean body mass is confirmed if the ALMI is < 5.5 kg/m^2^ in women or < 7 kg/m^2^ in men (Figures 3 and 4, respectively).

## LIMITATIONS OF DEXA

One of the main limitations of DEXA is the exposure to ionizing radiation, which, albeit low, can limit the performance of serial examinations. In addition, it can be difficult to position the patient correctly to perform the examination, especially if the patient is obese or has some functional limitation. Despite its low cost and broad availability in comparison with other imaging methods (especially CT and MRI), DEXA is not routinely used for all patients, being reserved for selected cases. In most patients, it is possible to assess nutritional status with more easily available, faster, lower-cost methods that can be performed in the office, including the measurement of anthropometric parameters, including skinfold thickness, and bioelectrical impedance analysis.

## CONCLUSION

The use of DEXA for the analysis of body composition provides important complementary information for assessing nutritional status, especially in patients at risk for sarcopenia. Despite its high accuracy and relatively low cost, DEXA is still not widely used in Brazil. It should be more extensively disseminated, so that more patients have access to and benefit from the use of this tool.
